# Surface-Tailored Zein Nanoparticles: Strategies and Applications

**DOI:** 10.3390/pharmaceutics13091354

**Published:** 2021-08-28

**Authors:** Ahmed M. Abdelsalam, Ahmed Somaida, Abdallah Mohamed Ayoub, Fahd M. Alsharif, Eduard Preis, Matthias Wojcik, Udo Bakowsky

**Affiliations:** 1 Department of Pharmaceutics and Biopharmaceutics, University of Marburg, Robert-Koch Straße 4, 35037 Marburg, Germany; ahmed.abdelsalam@pharmazie.uni-marburg.de (A.M.A.); somaida@staff.uni-marburg.de (A.S.); ayoub@staff.uni-marburg.de (A.M.A.); eduard.preis@pharmazie.uni-marburg.de (E.P.); matthias_wojcik@web.de (M.W.); 2 Department of Pharmaceutics and Industrial Pharmacy, Faculty of Pharmacy, Al-Azhar University, Assiut 71524, Egypt; fahdemam@azhar.edu.eg; 3 Department of Pharmaceutics and Industrial Pharmacy, Faculty of Pharmacy, Zagazig University, Zagazig 44519, Egypt

**Keywords:** zein, drug delivery, surface tailoring, active targeting, antigenicity

## Abstract

Plant-derived proteins have emerged as leading candidates in several drug and food delivery applications in diverse pharmaceutical designs. Zein is considered one of the primary plant proteins obtained from maize, and is well known for its biocompatibility and safety in biomedical fields. The ability of zein to carry various pharmaceutically active substances (PAS) position it as a valuable contender for several in vitro and in vivo applications. The unique structure and possibility of surface covering with distinct coating shells or even surface chemical modifications have enabled zein utilization in active targeted and site-specific drug delivery. This work summarizes up-to-date studies on zein formulation technology based on its structural features. Additionally, the multiple applications of zein, including drug delivery, cellular imaging, and tissue engineering, are discussed with a focus on zein-based active targeted delivery systems and antigenic response to its potential in vivo applicability.

## 1. Introduction

Proteins represent excellent candidates for drug and gene delivery due to their numerous advantages, e.g., biodegradability, ease of availability, high drug-binding capacity, and large surface area for drugs and cell binding. Therefore, the use of protein-based carriers in controlled and targeted drug delivery is exponentially increasing [1,2]. More interestingly, plant-derived proteins are gaining recognition in biomedical research because they are renewable and relatively inexpensive to produce. Such properties make plant proteins more reliable than animal-derived proteins [3].

Zein is an insoluble prolamin protein that is extracted from corn. It was approved in 1985 by the FDA as GRAS (generally recognized as safe) [4]. The utilization of zein was reported in organic coatings, for candy, fruit, and pills, in addition to its applicability as a platform for drug delivery and in tissue engineering [3,5].

So far, zein has been explored for multiple purposes, including oral delivery of proteins [6] and vegetable-derived materials [7], cell culture purposes [8], and vaccine delivery [9,10]. Additionally, zein has been engineered in versatile forms such as hollow nanoparticles [11], nanofibers [12], and micelles [13].

Zein has been reviewed as an excipient in drug delivery [14], as a solubility enhancer of poorly water-soluble drugs [15], as a scaffold in tissue engineering [16] and zein based-films [17], and as material for food and nutrition applications [2]. However, this review illustrates the structural features of zein and the possible surface alterations in the dispersion environment.

This work summarizes the interaction between zein and other macromolecules including phospholipids, polysaccharides, and proteins. Moreover, the combination of zein with other metallic-based nanomaterials for therapeutic or imaging purposes is discussed. It also highlights zein as an active targeting platform through either chemical or physical surface manipulation. Finally, an overview of its antigenic behavior, and of efforts to reduce the in vivo immune warning mechanisms, is included.

## 2. Structure of Zein

Zein is the major protein in corn, comprising up to 50% of the total corn protein. It is located primarily in the zein bodies distributed through the cytoplasm of corn endosperm cells [18]. The main characteristic of zein as a naturally occurring material is its solubility in hydroalcoholic solvents, in high alkali concentrations [16], and anionic detergents [19]. Such behavior was attributed to its unique structural characteristics, being rich in the nonpolar amino acid residues and deficient in the polar type residues.

**Figure 1:**
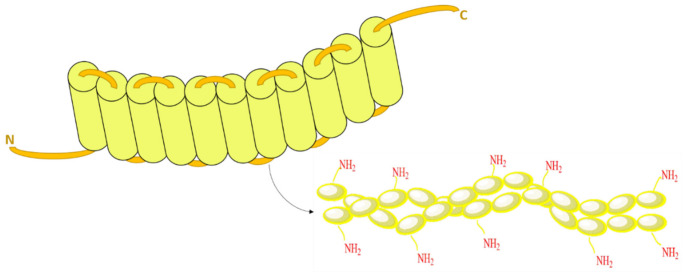
Hypothetical view of the tandem repeat units formed by single α- helix presented by cylinders and the abundant amine functionalities on the glutamine rich turns (loops) joining them.

Moreover, zein occurs in a mixture of peptides of variable molecular sizes, i.e., α, β, γ, and δ, as shown in Table 1 [20,21]. α-zein (19–22 kDa) is considered the most significant prolamin fraction, comprising around 80% of the total corn protein, with fewer histidine residues, arginine, proline, and methionine than the β-zein fraction [18]. A proposed three-dimensional structure was established through a small angle X-ray study of the α-zein [22], which assumed that each of tandem repeat units formed a single α -helix and were joined by glutamine-rich ‘turns’ or loops, as illustrated in (see Figure 1).

Due to the numerous nonpolar amino acid residues and the lack of water solubility, α-zein is a potentially valuable material for controlled and sustained release designs [23,24]. The amino acid glutamine over zein imparts a unique property upon its surface, being scattered with primary amine functionalities (see Figure 1). These turns could facilitate the integration of targeting probes onto the protein surface [25,26], or even complexation with other macromolecules [27,28].

## 3. Factors Affecting the Structure and Integrity of Zein Protein

The unique solubility criteria of zein were attributed to its structural architecture. The lack of lysine and tryptophan and the few arginine and histidine residues are the main differences between zein and other proteins [15]. However, its structural features may be affected by temperature and pH, which might be considered the leading influencers of its structure. In this part, we list some of the studies devoted to providing a clear picture of the effects of temperature and pH on the structure and conformations in solutions of zein.

### 3.1. Temperature Effect

Heat treatment of the native plant proteins is thought to bring about specific conformational changes in the protein architecture [29]. Sun et al. [30] reported structural change to zein protein following thermal treatment; physical, structural, thermal, and morphological characteristics were recorded. The heat treatment of zein dissolved in 70% ethanol at different temperatures (75 °C, 85 °C and 95 °C) for particular time intervals (15, 30 and 45 min) was studied. Atomic force microscopy, dynamic light scattering, and fluorescence spectroscopy, in addition to ultraviolet spectroscopy, circular dichroism, and differential scanning calorimetry, were employed to characterize the structural changes to the protein. This study revealed that by heat processing at 75 °C for 15 min, a narrower particle size distribution, an increase in α-helix and a decrease in β-sheet, and enhanced thermal stability were induced. In addition, thermal treatment at 85 °C for 30 min resulted in the formation of zein aggregates with larger size with an obvious increase in random coils and a distinct decrease in β-turn. Increased fluorescence intensity and change of the zein morphology from spherical to oval was also noticed.

**Figure 2:**
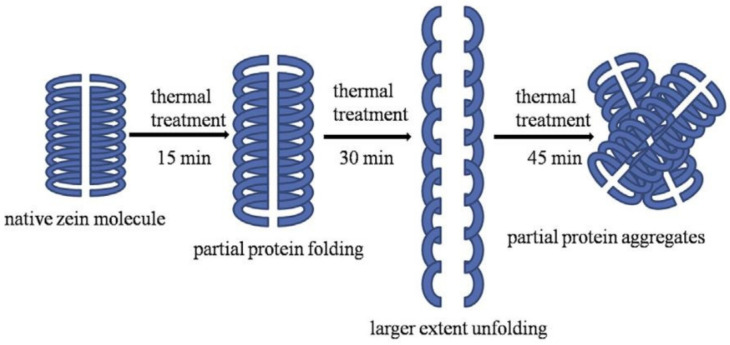
A schematic diagram of the potential mechanism of the three-step process involved in structural changes to zein following thermal treatment. Reproduced with permission from [30], ELSEVIER, 2021.

As shown in Figure 2, a three-step process was proposed to interpret these structural changes. The partial protein was folding at relatively low temperature for a short time (15 min). Then extensive unfolding was observed for a moderate temperature-time (30 min). Finally, the formation of partial protein aggregates was observed for an extensive time (45 min). However, similar structural findings were reported upon treatment with both heat and high-pressure homogenization [31].

Different studies have reported that the thermal treatment of zein nanoparticles in an aqueous environment affected the dispersion properties of the whole colloidal system through the rearrangement of the protein and the collapse of the colloidal structure [32]. Probably, with zein, as with many proteins, high-temperature treatment tends to cause denaturation and destruction of secondary and tertiary structures, causing protein agglomeration [33].

Despite the denaturing effect of heat on the structure of zein, recent reports utilized thermal treatment to obtain stable nanoparticles [34]. When a zein solution was injected into an aqueous phase of pH 4 at 70 °C, nanoparticles were acquired after stirring for 20 min and subsequent cooling. As a result of Raman analysis, the study concluded that the thermal treatment induced redistribution of the amino acid residues on the surface of the zein particles. Although the particle stability was further increased by electrostatic deposition of gum arabic or sodium alginate to the particle surface, heat-induced nanoprecipitation was the main driving force for the assembly of nanoparticles [34].

### 3.2. pH Effect

The pH of the environment in which drugs and food products are processed is an important parameter that influences their integrity and overall quality. The same is true for proteins, which are famous for being tremendously influenced by pH. In this respect, zein is one of the most well-known plant proteins to be affected by pH, having an isoelectric point of 6.2 [35], which gives rise to the tendency of zein to aggregate at near a neutral pH.

**Figure 3:**
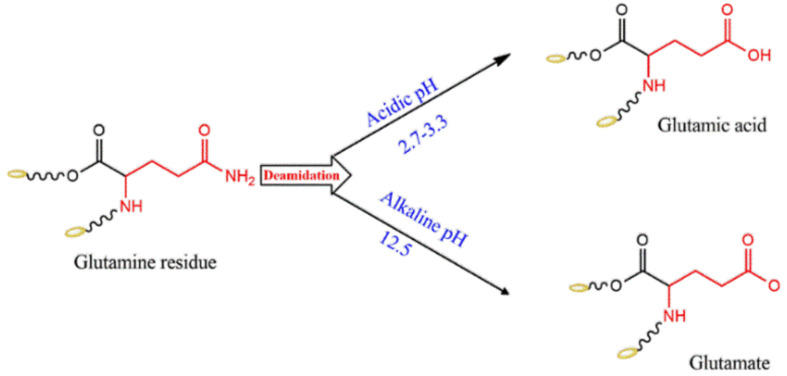
Possible deamidation products of zein at acidic and basic pHs.

The effect of acid and base on the structural, rheological, and antioxidant properties of the α-zein have been studied [36]. A solution of zein (0.1%) in 70% ethanol was allowed to react at room temperature for 24 h with different pH levels, i.e., neutral (6.5), two acidic levels (2.7 and 3.3), and two basic pH levels (10.5 and 12.5). The study results reflected a significant deamidation of the glutamine residue in the α-helix structure of zein at both the highly acidic and basic pHs (see Figure 3), as revealed by Fourier Transform Infra-Red (FT-IR) measurement. In contrast, the glutamine structure was retained at pH 6.5 and pH 10.5. Further confirmation with ξ-potential measurements indicated nearly no charge (−0.94 mV) at neutral pH and pH 10.5, but a negative potential at pH 2.7 and pH 12.5 due to deamidation into glutamic acid and glutamate, respectively [36].

Additionally, SDS-PAGE analysis revealed neither molecular weight change nor polymerization of the protein. Under the experimental conditions, the viscosity behavior of the 10% zein solution indicated a respective 6 and 10 times lower viscosity at the highly acidic and highly basic pHs than at the neutral pH. This was attributed to the reduced content of α-helix, β-sheets, and β-turns of the secondary structure of zein, which was in agreement with the FT-IR findings. The antioxidant properties of the acidic and basic treated zein were increased compared to those of the parent zein; this was attributed to the deamidation of glutamine into glutamic acid and glutamate, respectively, taking into account that antioxidant activity is positively imparted by glutamic acid-rich peptides, as applied in the acidic and basic treated zein [36].

The deamidation of α-zein by pH change could be utilized to design a specific zein based formulation. Accordingly, different deamidation procedures to obtain optimal emulsifying properties of zein have been reported [33]. Both acidic and basic enzymatic deamidation protocols have been employed to achieve maximum emulsion stability using base-treated zein [33].

Podaralla and Perumal [35] utilized an alkaline pH to control the nanoprecipitation of zein protein in neutral pH (7.4), and the stability of the zein nanoparticles was extensively studied and characterized. More recently, curcumin was encapsulated into deamidated zein nanoparticles through basic deamidation treatment. This protocol enhanced the solubility, stability, and antioxidant activity of the curcumin [37].

In addition to the deamidation hypothesis in the highly acidic or basic environment, zein molecule tends to carry an overall positive potential in slightly acidic pH values as well in pure water due to protonation of the free primary amine functionality of the glutamine residue. This feature was utilized to form complex structures with other macromolecules [38]. However, zein tends to aggregate at near-neutral pH, as this is quite close to its isoelectric point [35,39].

## 4. Zein Nanocomposite-Based Systems

The interaction of zein with various molecules yielded a massive dose of nanocomposite materials, which have been recognized as reliable systems for drug delivery applications. This section lists some of the reported zein-based nanocomposite complexes employed either in food or drug delivery applications.

### 4.1. Zein Phospholipid Complex

Dai et al. [40] studied the interaction between zein and the low molecular weight surfactant “lecithin” in an ethanol-water mixture, and the coprecipitated zein-lecithin nanoparticle dispersion. The effects of pH, temperature, salt concentration, and lecithin amount on the stability of the colloidal zein nanoparticles were addressed. The findings revealed a significant change in the secondary structure of zein, accompanied by improved thermal and salt stability with increased lecithin amounts in the system. A critical zein-to-lecithin ratio of (5:1) was proposed to translate the behavior of the system. This assumption revealed that the tendency for aggregation of the zein and lecithin composite colloidal nanoparticles at a lower lecithin concentration (with a zein-to-lecithin mass ratio ≤ 5:1) was due increased interaction with the higher proportion of lecithin. The author claimed that a continual increase of lecithin might lead to an excessive number of lecithin molecules covering the zein-lecithin composite surface, which would generate a vesicle-like structure. Hence, lecithin inhibits the aggregation and improves the thermal and salt stability of the composite colloidal nanoparticles.

**Figure 4:**
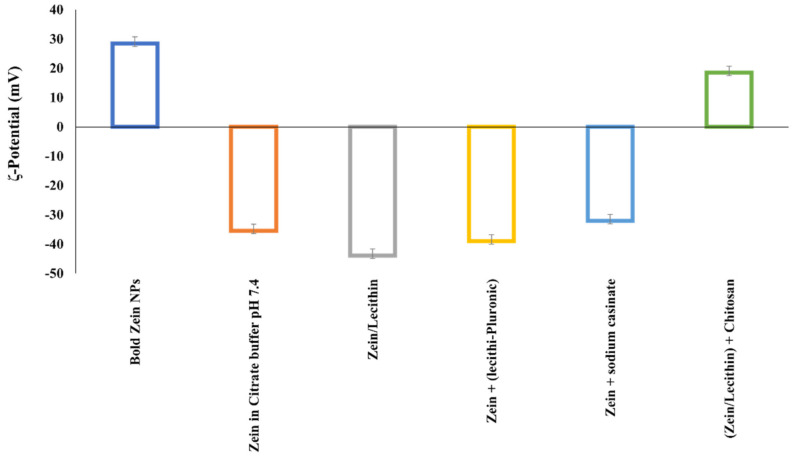
Change in zein surface potential by changing the dispersion environment.

Additionally, Podaralla and Perumal [41] reported similarly enhanced stability of the zein-lecithin nanocomposite in the presence of pluronic F68. A stable nanocomposite system was obtained and employed as a platform for encapsulating 6,7-dihydroxy coumarin (DHC) as a model hydrophobic compound. Besides the higher stability against change in the ionic strength, the formulation buffer system indicated that the employment of a citrate buffer pH 7.4 imparted higher stability upon the system compared with PBS 7.4 during the lyophilization process. Moreover, the use of pluronic F68 as a nonionic surfactant to strengthen the stability of zein-lecithin nanocomposite systems has been reported in the literature [42]. It was presumed that, in addition to the shielding effect of the nonionic pluronic molecule, the citrate buffering system might be opsonized by the zein nanoparticle surfaces. Hence, an ionic interaction between the positively-charged zein and the negatively charged citrate might occur. This type of zein interaction with several coating materials accounts for the significant change in ζ-potential in different dispersion environments, as shown in Figure 4.

### 4.2. Zein Protein Complex

Forming a protein–protein complex is a unique approach to obtain a stable zein nano complex system with high reliability in diverse delivery applications.

Solvent-free, self-assembly of zein and the sodium salt of milk protein casein has been reported [43]. A change of the pH from 11.5 to 7 triggered the macromolecule self-assembly into nanoparticles. This led to as low as 100 nm nanoparticle diameters, with considerable stability and dispersibility. Moreover, the use of caseinates as zein stabilizers was found to improve the solubility and bioavailability of simvastatin [44]. Data have also shown that drug solubility and bioavailability were enhanced. Even after treatment with several cellular uptake inhibitors, the cellular uptake of zein-caseinate nanoparticles in colorectal CaCo-2 cells was also increased [45].

**Figure 5:**
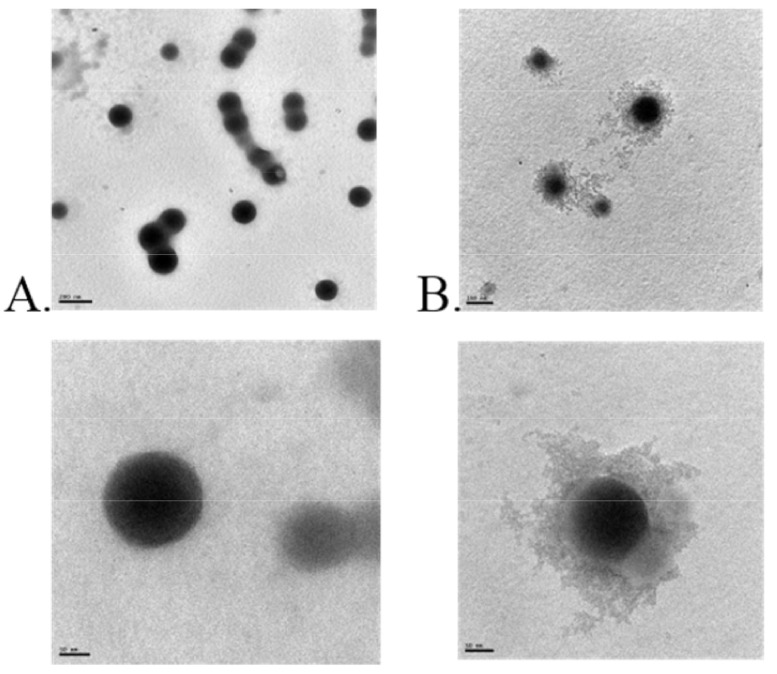
Transmission electron microscopic images of zein-casein (**A**) and zein-lactoferrin (**B**) nanoparticles. The lower panel shows the individual nanocarriers at a higher resolution (scale bar 50 nm). The scale bar in the upper panel is 200 nm for ZC nanoparticles (**A**) and 100 nm for ZLF nanoparticles (**B**). Reproduced with permission from [46], American Chemical Society, 2017.

Lactoferrin, a milk protein that belongs to the transferrin family and which is of high value for infants as an iron transporter, was utilized to coat the surface of zein to form a zein-lactoferrin core-shell complex [46]. As confirmed by transmission electron microscopy, compared to caseinate, lactoferrin produced a large shell over the surface of the zein (see Figure 5) which was attributed to the considerable molecular weight of lactoferrin (about 80 kDa).

Ji et al. [47] employed carboxymethylated short-chain amylose (CSA) to coat insulin-loaded zein nanoparticles where insulin and zein were co-injected into an aqueous CSA solution. Using zein/natural polymer nanoparticles can improve the physical stability of zein nanoparticles over a wide range of pH, temperature, and ionic strength [48]. The CSA coat raised the stability of the zein/insulin mixture. The fluorescence quenching of tyrosine and tryptophan residues of zein and insulin at increased concentration of the CSA supported the proposed complex formation between zein/insulin and CSA. Additionally, FT-IR investigations indicated nanoparticle formation by hydrogen bonding and hydrophobic interactions between zein, insulin, and CSA. The entrapment efficiency was 45.8% in insulin-loaded zein NPs, which increased to 90.5% after the CSA was added. Presumably, CSA interacted with free insulin by electrostatic interaction, increasing the entrapment efficiency of the insulin.

Recently, a study evaluated the pharmacokinetic profile of orally administered insulin-loaded zein nanoparticles coated with a poly(anhydride)-thiamine conjugate [6]. Glycemia was found to be reduced by 60% in animals, and the bioavailability of the nano-encapsulated insulin was 13.5%. Compared with a subcutaneous insulin formulation, zein nanoparticles provided a maximum of insulin 4 h postadministration, and relative oral bioavailability was 5.2% in serum.

### 4.3. Zein Polysaccharide Complex

Polysaccharide-based materials were found to form stable coverings over the surface of zein to obtain a diverse spectrum of nanocomposite systems [39,49,50]. The commonly known polysaccharide chitosan was reported to form a surface coating on top of zein nanoparticles [51,52]. Isothermal titration calorimetry revealed a spontaneous exothermic interaction process between zein and chitosan [53]. Hence, the more intense the interaction between zein and chitosan, the greater the number of yielded spherical particles. Moreover, at pH 4, the zein and chitosan composite revealed a small particle size (<200 nm), stable ξ-potential (+50 mV), high encapsulation efficiency of the model drug curcumin (95%), and a relatively low release rate profile. It was concluded that zein-chitosan coacervate at a moderately acidic pH value could be employed as potential oral delivery platform.

The valuable role played by chitosan in enhancing the stability of zein nanoparticles was studied by Park et al. [51], who observed an increase in retinol entrapped in zein from around 65% to more than 80% after overlaying chitosan onto zein nanoparticles. The chitosan coating resulted in a decrease of the initial burst release of retinol. Moreover, retinol exhibited relatively higher photochemical stability against UV light following encapsulation in chitosan nanoparticles.

The fabrication of zein-chitosan complex nanoparticles enhanced the cellular uptake and intracellular antioxidant activity of quercetin in colorectal adenocarcinoma Caco-2 and human hepatocyte carcinoma HepG 2 cells, respectively [54]. The stability of the incorporated quercetin was enhanced at pH 7.4 with a noticeable increase in UV and heat stability. Alternatively, carboxymethyl chitosan (CMCS), a structural analog of chitosan, was reported to coat the surface of zein nanoparticles incorporating vitamin D3 in the presence of calcium as a crosslinker [55]. A significant improvement in the encapsulation efficiency (87.9%) combined with photostability against UV was recorded for the CMCS-coated zein compared to the naked one (52.2%).

Numerous studies have reported the use of zein/CMCS nanoparticles to stabilize various active pharmaceutical and food products [55,56]. The idea behind the addition of CMCS is that zein alone maybe not enough to attain optimal protection. Accordingly, zein nanoparticles were coated with chitosan and its analogs to enhance their physicochemical properties, stability, and release profile [57]. On the other hand, upon incubation of zein at 37 °C for 24 h, it tends to denature, resulting in nanostructure crumbling, and ultimately precipitation. However, the chitosan coating probably slows down the denaturation rate by forming a protective sheath. Zein/CMCS nanoparticles can protect bioactive compounds such as indole-3-carbinol and 3,3′-diindolylmethane for four days at 37 °C [58]. In addition to their thermal stability, zein/CMCS nanoparticles can protect these phytochemicals against light-induced degradation. The protection against light can be attributed to the aromatic rings in zein that absorb UV light [58].

More interestingly, Farris et al. [59] used zein and chitosan as a sole system for oral DNA delivery. Their hypothesis was based on bypassing the harsh stomach conditions, i.e., by utilizing zein as a gastroprotective coat covering the surface of DNA-loaded chitosan nanoparticles. The zein coat rapidly decomposed upon reaching the intestine, ultimately releasing DNA/chitosan nanoparticles that could reach their target.

Alginates were also tested as surface stabilizers for zein nanocarriers [27,60]. A nanocomposite with zein as the core and alginate as the surface shell was reported to impart heat stability, lower agglomeration behavior over a wide pH range, and optimized physicochemical characteristics at slightly acidic pH [60]. The zein-alginate complex system was utilized to encapsulate the acid-sensitive superoxide dismutase (SOD) [27] thanks to the acid resistance feature of zein that is thought to protect SOD from extreme gastric acidity and conserve its superoxide scavenging properties. On a cellular level, reactive oxygen species (ROS) in Caco-2 cells were reduced within 4 h, an effect that saved up to 89% of the cells from the toxic superoxide levels.

The use of the acid-resistant property of zein was further exploited for the oral delivery of amino acids [61]. Selenomethionine, an essential amino acid with low oxidation stability and narrow therapeutic index [62], was encapsulated into chitosan nanoparticles coated with zein for oral delivery [63]. The developed selenomethionine-entrapped nanoparticles were found to be nontoxic to intestinal and liver cell lines. Additionally, accelerated thermal stability studies indicated good stability under normal storage conditions (23 °C). A cumulative release of about 60% was obtained over 6 h in simulated gastrointestinal and intestinal fluid conditions. However, these findings illustrated the beneficial protective role of zein to incorporated proteins in terms of overcoming the harsh gastric acidity.

Chondroitin sulfate was also utilized as a surface coating macromolecule for sorafenib carrying zein nanoparticles [64]. The obtained core-shell nanocomposite was employed for the delivery of sorafenib to gastric cancer cell lines. The spherical shape and the controlled release of the system indicated its potential applicability in systemic drug delivery. Recently, H. S. Lee et al. [65] developed chondroitin sulfate/zein hybridized nanoparticles for the targeted delivery of docetaxel. The nanoparticles exhibited a CD44 mediated uptake in PC-3 prostate cancer cells and about a 2.8-fold reduction in the IC_50_ of docetaxel compared to the free compound. Moreover, the in vivo pharmacokinetic profile established in PC-3 xenograft mice indicated a 9.5-fold longer terminal half-life as compared to free docetaxel. Negligible systemic toxicity of the nanoplatform was obtained in comparison to Taxotere, suggesting that zein/chondroitin sulfate nanoparticles could be a promising nanoplatform for targeted cancer therapy.

Furthermore, other complex shells such as alginate/chitosan complex [50], caseinate/alginate complex [66], pectin/caseinate complex [67,68], and alginate/gelatin complex [69] have been investigated as zein surface coatings. These strategies were employed to increase physicochemical stability and biocompatibility, and to achieve higher encapsulation efficiency. Therefore, a range of versatile nanocomposite systems with different potential food and drug delivery applications was obtained.

## 5. Applications in Drug Delivery

Besides the safety of zein in food applications, it is also an FDA-approved material for biomedical applications [4]. Therefore, zein-based carriers have been utilized for the delivery of several active compounds, including hydrophilic and hydrophobic drugs [66,70], vitamins [71], antigens [72] and dyes [73].

Moreover, its amphiphilic properties, film-forming capability, and biodegradability render zein a highly sought after material for the production of packaging materials [74] and as tissue engineering scaffolds [16], in addition to being a reliable, biodegradable platform for biosensors, including microfluidic devices [75]. Furthermore, zein protein-based carriers have the advantage over the animal-derived proteins of being devoid of the possibility of causing zoonotic disease transmission [76]. In the following paragraphs, we summarize some zein-based drug delivery systems and their applications in different delivery fields.

### 5.1. Phytochemical Drug Delivery

Zein has been extensively used to enhance the solubility of curcumin [69,77]. It has been reported that the hydrophobic interaction between zein and curcumin leads to an 8200-fold increase in the water solubility of the latter. Additionally, this complexation promotes the chemical stability of curcumin during storage [78]. Recent work reported using a pH-driven nanoprecipitation method to achieve the encapsulation of large amounts of curcumin into rhamnolipid-zein composite nanoparticles [79]. The developed rhamnolipid-zein nanocomposite protected the loaded curcumin for up to one month of storage at 25 °C and 37 °C. Additionally, caseinate/sodium alginate dual-coated zein nanoparticles have been utilized to incorporate curcumin [66], a strategy that enhanced photostability, water solubility, and the antioxidant properties of curcumin with controlled release behavior in simulated gastric fluid.

Quercetin (a natural antioxidant flavonoid) was also encapsulated via nanoprecipitation into zein nanoparticles at acidic pH in the presence of soluble soybean polysaccharides [80]. The soybean polysaccharide coating increased the stability of the zein nanoparticles under relatively high ionic strength conditions and elevated temperature. Interestingly, increased encapsulation efficiency, photochemical stability, and antioxidant activity were achieved after modifying quercetin-loaded zein with the soluble soybean polysaccharide.

Alcohol-soluble quercetgetin, a flavonoid with known antioxidant and hepatoprotective effects, was incorporated into zein nanoparticles [81]. More interestingly, Chen et al. [38] reported a zein-hyaluronic acid binary complex nanocomposite for the encapsulation of quercetgetin. The zein-hyaluronic acid revealed a uniquely new nanocomposite with adequate thermal stability and controlled release features of quercetgetin, coupled with a prominently high encapsulated amount of the active flavonoid.

Moreover, to preserve its beneficial properties, lutein, an antioxidant carotenoid prone to degradation in water, UV light, and heat, was entrapped in zein nanoparticles stabilized by lecithin and pluronic F127 surfactants. The system could retain lutein away from the harsh conditions, resulting in better protection against thermal and UV decomposition compared to emulsions [82].

Nevertheless, several reports have described the use of zein-based carriers to encapsulate various vegetable-derived active compounds [82,83,84,85].

### 5.2. Essential Oil Delivery

**Figure 6:**
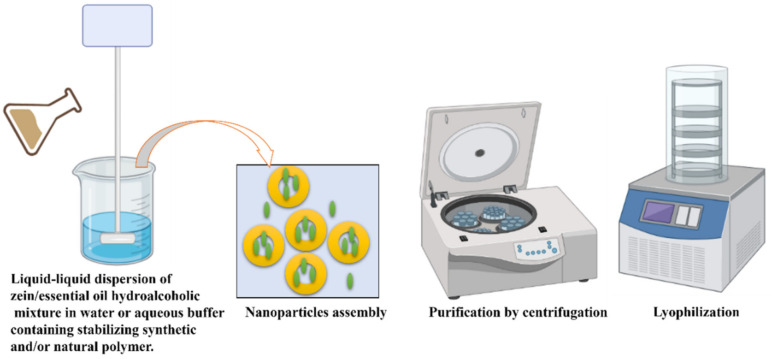
The encapsulation of essential oils into zein by the liquid–liquid dispersion approach and the separation and lyophilization of the formulated nanoparticles.

Many encouraging observations have shown that zein nanoparticles could be a promising approach for the effective delivery of essential oils (EOs) by preserving their antimicrobial action, promoting stability, or masking unpleasant flavors. In general, essential oils can assemble into the core of the amphiphilic zein by employing liquid–liquid dispersion procedures, as shown in Figure 6.

Peppermint oil was incorporated into zein nanoparticles using propylene glycol as a solvent and gum arabic as a stabilizer [86]. The nanoparticles were stable over a pH range of 3.0–8.0, which was attributed to both the electrostatic and hydrophobic interactions produced by gum arabic adsorption at the surface.

In another study, thymol was encapsulated into zein using sodium caseinate as a stabilizer via a liquid–liquid dispersion method [87]. Work was extended to study the effect of chitosan coating via layer-by-layer electrostatic deposition onto caseinate-coated zein nanoparticles; however, this coating ended in higher entrapment efficiency. Furthermore, chitosan improved the redispersibility of lyophilized nanoparticles in water due to electrostatic stabilization. The results indicated that the nanoparticles enhanced and prolonged the antimicrobial activity of thymol against *Staphylococcus aureus*, an effect that was found to be superior to that of free thymol.

**Figure 7:**
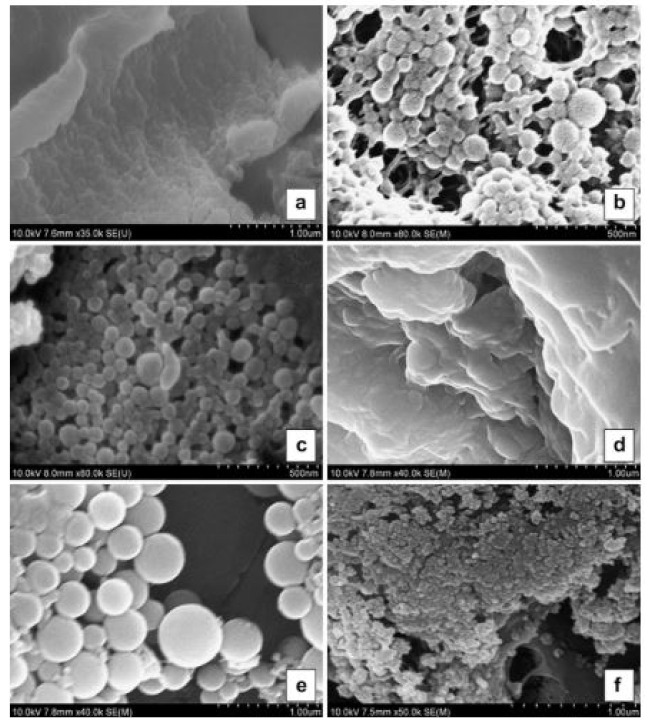
SEM images for samples encapsulating EOs under different pH conditions after lyophilizing. (**a**–**c**) encapsulated thymol under pH = 4, 6.5 and 10, respectively. (**d**–**f**) encapsulated carvacrol under pH = 4, 6.5 and 10, respectively. Reproduced with permission from [88], ELSEVIER, 2021.

Similarly, thymol and carvacrol were incorporated into zein nanoparticles using a liquid–liquid dispersion method under different pH conditions, resulting in spherically-shaped zein nanoparticles (see Figure 7) with an entrapment efficiency higher than 50%. It was found that the zein nanoparticles led to a 14-fold increase in the solubility of the essential oil while maintaining their antioxidant features. Interestingly, the nanoparticles exhibited 1.8 log reductions in *Escherichia coli* growth; presumably, *Escherichia coli* digests zein nanoparticles, causing rapid disengagement of the entrapped oils [88].

### 5.3. Cytotoxic Drug Delivery

The amphiphilic characteristics of zein could be utilized to improve the bioavailability of many hydrophobic cytotoxic drugs, as well as to sustain the release of other hydrophilic candidates, and hence, to reduce the side effects associated with multiple drug dosing.

The low molecular weight antineoplastic agent 5-fluorouracil (5-FU) was encapsulated into zein nanoparticles for liver targeting in a mouse model [70]. The optimized formulation had an encapsulation efficiency of up to 60% with sustained release behavior. The nanoparticles were found to accumulate in the liver following intravenous injection, with an increased targetability of more than 31%. The study discussed the potential applicability of zein nanoparticles for liver targeted drug delivery via the intravenous route.

The anthracycline antibiotic doxorubicin was encapsulated into zein nanoparticles [89]. The developed system exhibited uniform, spherically-shaped particles with sustained, pH-responsive release of doxorubicin. An in vitro study on cervical cancer HeLa cells revealed higher toxicity and the antiproliferative effect of zein-doxorubicin nanoparticles compared to free doxorubicin. The cellular uptake behavior toward the doxorubicin-loaded zein nanoparticles was controlled by macropinocytosis based on the results obtained after the treatment of cells with the corresponding uptake inhibitors.

A recent study demonstrated the use of hydroxyapatite/zein nanocomposite as a carrier for doxorubicin hydrochloride [90]. The results indicated good colloidal stability. Additionally, good dispersibility after lyophilization with a pH-responsive release of doxorubicin HCl was attained. In vitro investigations revealed higher cellular uptake and cytotoxicity against mouse breast tumor 4T1 cells. Moreover, reduced cardiotoxicity and enhanced bioavailability were achieved in vivo.

Zein nanoparticles have also been used as a platform in combination chemotherapeutics for the delivery of vorinostat and bortezomib in prostate cancer [91]. The developed nanoparticles exhibited an entrapment efficiency above 60% combined with controlled release behavior. The system showed higher release under acidic compared to basic conditions due to lower stability and a conformational change in the zein structure in an acidic pH. This preferential release is potentially useful in cancer treatment delivery via the disassembly of the drug in acidic, cancerous tissues while avoiding premature release in the bloodstream and providing higher selectivity and fewer unwanted side effects. Additionally, cytotoxicity results presented a beneficial synergism in prostate cancer cell lines. Consistent with these results, confocal laser scanning microscopy (CLSM) revealed a higher uptake of zein nanoparticles by prostate cancer cells. This combined therapeutic regimen produced higher apoptosis than individual treatment. Finally, this combination imparted a higher antitumor effect in animal models, as indicated by lower tumor volume. This effect was ascribed to the ability of zein nanoparticles to target cancer cells and control drug delivery [91].

In a study by Jain et al. [84], the water solubility, chemical stability, and oral bioavailability of beta carotene (βC) were enhanced by incorporation into zein nanoparticles. The IC_50_ value of βC on the MCF-7 breast cancer cells decreased from 119.75 µg/mL to 89.13 µg/mL when encapsulated in zein nanoparticles. More importantly, βC enhanced the cytotoxic effect of methotrexate (MTX) on breast cancer MCF-7 cells where the βC nanoparticles + MTX attained an IC50 of 6.34 µg/mL, compared to 15.72 µg/mL for MTX alone. Next, CLSM indicated a marginally higher uptake of βC nanoparticles than free βC, which could be explained by enhanced endocytosis due to the nanostructures. Furthermore, pluronic F-68 incorporated with nanoparticles suppresses the P-glycoprotein efflux pump and improves the retention of nanoparticles in the cytoplasm. However, the highest apoptosis was accomplished when the antioxidant βC was codelivered with MTX. Compared to free βC, the in vivo pharmacokinetics analysis yielded a 2.3- and 2.7-fold increase in maximum plasma concentration (C_max_) and area under the concentration-time curve (AUC), respectively, for the zein-loaded βC. The authors credited these findings to the enhancing effect of the nanosized formulation and the mucoadhesive properties of zein that increase nanoparticle residence time in the gut mucosa. Finally, the co-administration of βC nanoparticles and MTX exhibited a marginal antitumor effect in the animal model, revealing a synergistic effect of βC and MTX. Surprisingly, free radical scavenging and the retarded oxidative stress of βC-nanoparticles improved MTX-induced renal and hepatotoxicity.

## 6. Zein in Cellular Imaging and Tissue Engineering

Owing to its biocompatibility and surface functionality, zein has been employed as a drug delivery carrier as well as in tissue engineering [8,9,72,92]. Furthermore, its solubility features have made it valuable in sustaining the release of many hydrophilic and hydrophobic active compounds by forming zein-based microspheres, solid dispersions, and nanoparticles [15]. Herein we summarize some of the most recent literature that has employed zein in cellular and tissue imaging and tissue engineering.

### 6.1. Bioimaging

**Figure 8:**
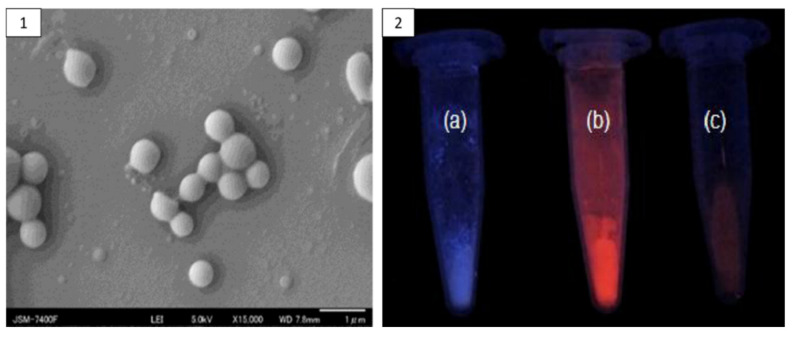
SEM Image of zein nanoparticles (**1**), and photograph of fluorescent (**a**) zein nanoparticle, (**b**) QD, and (**c**) zein QD under UV illumination (**2**). Reproduced with permission from [93], IOPSCIENCE, 2012.

The application of zein-based nanocarriers for simultaneous drug delivery and bioimaging has been discussed in a few literature reports. Aswathy et al. [93] designed a quantum-dot (QD)/zein nanoparticles using a liquid–liquid dispersion technique with ZnS-Mn QD alongside zein in 90% isopropanol and dropped into water at room temperature to yield spherical luminescent nanoparticles (see Figure 8). Subsequently, 5-fluorouracil was used in their study as a model cytotoxic agent. The developed nanoparticles were tested for biocompatibility and cytotoxicity in mouse fibroblast L929 and breast MCF-7 cells. The results showed that more than 90% of the cells were viable after incubation with bare zein nanoparticles. Comparatively, more than 80% of cells were viable after incubation with zein-QD, indicating relatively similar biocompatibility. On the other hand, a considerable reduction in cell viability was observed after treatment of the cells with the 5-FU-loaded zein-QD nanoparticles. Besides the efficient cellular imaging of the QD/zein nanosystem in vitro, the nanoparticles were presumed to have potential applicability in targeted drug delivery and as a fluorescent platform.

**Figure 9:**
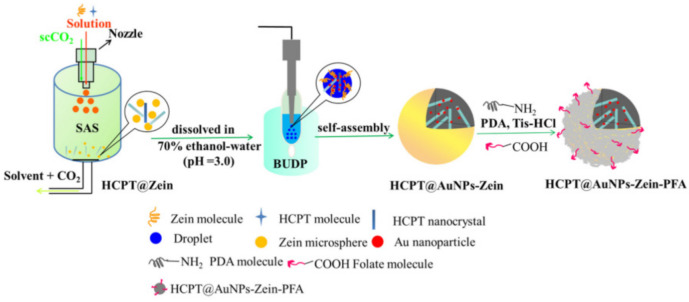
Schematic diagram of the preparation of HCPT@AuNPs-Zein-PFA NCs. Reproduced with permission from [94], ELSEVIER, 2021.

**Figure 10:**
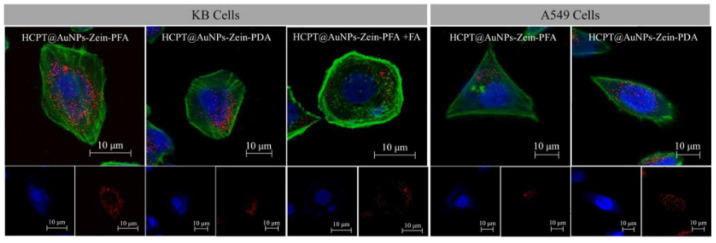
CLSM images of KB and A549 cells treated with HCPT-loaded NCs (HCPT@AuNPs Zein-PFA NCs in the presence of excessive free folate) at 37 °C for 1 h. The plasma membranes of cells were stained green by Alexa Fluor 488 phalloidin. Their nuclei were stained blue by DAPI. Nanoparticles were labeled red with cy5. Reproduced with permission from [94], ELSEVIER, 2021.

H. Wang et al. [94] developed a nanocomposite delivery system by applying modified nanoprecipitation procedures of hydroxycamptothecin nanocrystals into zein microspheres co-incorporating gold nanoparticles as an imaging label (see Figure 9). Subsequently, polydopamine folic acid was added as a targeting probe to yield the hydroxycamptothin gold nanoparticles zein folate assembly (HCPT@AuNPs-Zein-FA). Despite the targeting ability of the nanocomposite, the design of HCPT@AuNPs-Zein-PFA with a AuNP core was advantageous for the uptake of nanocomposites in cells (see Figure 10) and tissues. It could be quantified by detecting the Au content using inductively coupled plasma atomic emission spectroscopy (ICP-AES) with high detection sensitivity. Thus, hydroxycamptothin accumulation can be easily calculated by detecting Au content since the ratio of each component in the HCPT@AuNPs-Zein-PFA nanocomposites was determined. It was suggested that this composite system would make intracellular quantification and localization of nanocomposites much more accurate and convenient.

### 6.2. Tissue Engineering

Zein has been reported as a widely-used material in tissue engineering [8]. Hadavi et al. [95] rationalized the use of zein as a platform for osteoinduction in mouse myoblast C2C12 cells in vitro. The bone morphogenic protein-6 (BMP-6), a peptide growth factor in bone formation and the regeneration of high osteogenic activity with a short half-life, was incorporated into zein nanoparticles via a liquid–liquid phase separation approach. The study revealed encapsulation of 72% and sustained release behavior of the loaded peptide, in addition to great biocompatibility of the peptide-loaded zein, with cell viability of more than 90% after 48 and 96 h incubation. Moreover, a significantly higher level of alkaline phosphatase, as an osteocyte marker, was observed in the C2C12 cells treated with BMP-6-loaded zein nanoparticles compared with the blank zein nanoparticles on day 14 following incubation. The real time quantitative polymerase chain reaction (RT-qPCR) confirmed that the BMP6 peptide-loaded zein nanoparticles significantly promoted Runx2 expression compared to blank nanoparticles, which indicated that the BMP pathway had been activated. It is noteworthy that Runx2 is a frequently described gene for osteoblast differentiation which regulates the differentiation of mesenchymal progenitor cells to pre-osteoblasts and induces the expression of noncollagenous proteins such as osteocalcin (OCN) and osteopontin (OPN); however, the study suggested that the BMP6 peptide-loaded zein nanoparticles may be a good candidate in bone regenerative medicine.

More recently, zein electrospun fibrous scaffolds crosslinked with trimethylolpropane triglycidyl ether were reported for bony tissue regeneration [96]. The in vitro assessment of the cryopreserved murine preosteoclast bone progenitor cells (MC3T3-E1) revealed a significantly high level of cellular growth and differentiation, irrespective of the osteoinduction factors in culture media. Increased alkaline phosphatase activity, mineralization, and early-stage upregulation of Runx2 gene expression were also recorded.

In addition to the previously described applications of zein, Table 2 summarizes some of the latest zein-based systems with diverse food and drug delivery applications.

## 7. Surface Modified Zein Nanoparticles for Active Targeted Drug Delivery

The development of drug and gene delivery transporters labeled with active targeting probes is a new method by which to deliver the loaded cargo precisely into the desired location in a biological system [127]. Hence, a significant proportion of the payload tasked with provoking a pharmacological response should reach the target site with reduced leakage into other sites, thereby reducing side effects that would occur with the use of bare, nontargeted particles or with the drug alone.

### 7.1. Zein-Folic Acid

Zein is considered an ideal candidate platform for active targeted drug delivery due to the abundant amine functionality in its structure. In this respect, numerous research projects have used folic acid (FA), a known essential water-soluble vitamin required for cell growth and division, to label zein protein via chemical manipulation [26]. Folic acid specifically binds to the folate receptors on the surfaces of various types of cancer cells and facilitates the transfer of folate-decorated nanoparticles through receptor-mediated endocytosis.

Liu et al. [128] explored the targeting capabilities of folate-decorated zein nanospheres on folate receptor overexpressing cancer cells. FA was coupled to zein through amide coupling chemistry between the activated acid and the amine functionalities of zein. In an earlier study, a novel built-in ultrasonic dialysis process (BUDP) was employed to fabricate zein microspheres [129]. The strategy was further applied to encapsulate 10-hydroxychamptothecin [130]. A combinatorial methodology involving the supercritical antisolvent and this built-in ultrasonic dialysis approach was applied to obtain FA-zein nanoparticles carrying 10-hydroxychamptothecin nanocrystals [94]. An assessment of the cellular uptake on folate receptor positive cervical cancer Hela cells and folate receptor negative alveolar cancer A549 cells indicated a higher accumulation of the FA-labeled nanospheres in the former. Additionally, the in vitro cytotoxicity of the FA-zein-loaded 10-hydroxychamptothecin nanocrystals of the same concentrations revealed prominently lower viability in the Hela cells than in the A549 cells.

Folic acid was chemically coupled to zein and utilized for active targeting, either as a sole carrier [26] or complexed with another nanosystem, such as superparamagnetic iron oxide nanoparticles [131]. In contrast, Hou et al. [132] studied the noncovalent conjugation between zein and folic acid. The study claimed that folic acid tends to assemble over the surface of zein via reversible ionic hydrogen bonds. The two carboxylic acid groups and the sole amino group of the folic acid molecule drove this type of assembly. This type of protein-acid conjugate was employed to incorporate doxorubicin hydrochloride to form a bilayer zein-folic acid nanocarrier, which was found to sustain the in vitro release of doxorubicin, in addition to providing enhanced therapeutic efficacy in vivo.

Another study reported paclitaxel delivery to folate receptor-expressing KB cells via a PEGylated folic acid-zein nanoconjugate system [133]. FA was chemically coupled to polyethylene glycol by an amide linkage before being reacted with already made paclitaxel zein nanoparticles to yield a folic acid-PEG-zein nanoconjugate with incorporated paclitaxel (PTX). The in vitro cell viability of KB cells treated with PTX/zein-FAs was the lowest, and the IC50 value was also observed to be lower than in cells treated with free PTX and PTX/zein nanoparticles, which indicates increased potency. A KB-tumor bearing xenograft mouse model was employed for an in vivo biodistribution study using encapsulated Cys 5.5 as a fluorescent dye. The fluorescence signal in the tumor area of the Cy5.5/zein-FA-treated group was about two times higher than that of Cy5.5/zein NPs after a treatment of 24 h, thus proving the accumulation of nanoparticles in the tumor area due to the targeting effect of the nanoparticles. Moreover, folate-targeted NPs significantly promoted the antitumor efficacy of PTX in folate receptor overexpressing cancer cells. The significant inhibition in tumor growth rate in the PTX/zein-FA-treated group compared to free PTX and nontargeted NP treatment groups was correlated to the folate targeting capabilities.

### 7.2. Zein-Lactoferrin

The use of lactoferrin, a cationic, mammalian, iron-binding glycoprotein, and LDL receptor binding probe, was investigated as a targeting ligand for breast cancer cells where the LDL receptors were overexpressed [134,135].

El- Lakany et al. [42] investigated the targeting ability of lactoferrin-decorated zein nanospheres fabricated to co-encapsulate both exemestane (EXM), an irreversible steroidal aromatase inhibitor (AI), and luteolin, a flavone with reported anticancer and EGFR- inhibitory actions. The zein nanospheres were fabricated and stabilized with lecithin, and pluronic F68. The positively-charged lactoferrin was adsorbed on the negatively-charged zein nanoparticle surface via electrostatic attraction. MCF-7 breast cancer cells and 4T1 murine cells were employed to investigate in vitro cytotoxicity. A decrease in cell viability with lower IC_50_ was observed in the lactoferrin labeled formula in comparison to the free exemestane/luteolin.

Sabra et al. [136] managed to chemically synthesize zein-lactoferrin copolymer micelles through a carbodiimide coupling reaction to target breast cancer. The developed polymeric micelles were utilized to encapsulate rapamycin (RAP) and wogonin (WOG), two hydrophobic materials with known anticancer activities [137,138]. The combinatorial delivery of these two drugs was thought to synergistically inhibit mTOR/PI3K/AKT pathways by bypassing the feedback survival activation pathway induced by RAP monotherapy. Glutaraldehyde was employed as a crosslinker to improve micellar stability. Furthermore, uncrosslinked and glutaraldehyde crosslinked zein-lactoferrin micelles were tested against MCF-7 cells invitro and Ehrlich’s mammary tumor-bearing mouse model in vivo. The results revealed that glutaraldehyde-crosslinked micelles exhibited enhanced cellular uptake as well as enhanced in vitro cytotoxicity against MCF-7 breast cancer cells. Moreover, the tumor volume reduction, suppression of CD-1 pathway required for tumor proliferation, and inhibition of the tumor angiogenesis factor VEGF were observed for the crosslinked micelles in contrast to the uncrosslinked ones.

## 8. Antigenicity of Zein

Despite the extensive use of zein in delivery applications, few investigations of its in vivo antigenicity have been published. This antigenicity may contribute to provoking immune response and the formation of antizein antibodies, thus hindering its in vivo application.

In this regard, the effect of size, dosage, and route of administration on the in vivo antigenicity of zein nanoparticles in BALB/c mice was explored in a single study by F. Li et al. [139]. This study was based on the fact that the parenteral administration of foreign materials tends to provoke an antigenic response, primarily at the injection site. Freshly prepared zein nanoparticles obtained by the antisolvent precipitation method were administered into different groups of female BALB/c mice via two separate administration routes, i.e., the intramuscular (IM) and the subcutaneous (SC) injection. Through the two routes, a dose of 200, 600, or 800 microgram/mouse was administered at weeks 0, 1, and 3. Fifty weeks after the first inoculation, treatment groups were challenged with zein nanoparticles of the same previously administered doses. Blood sera were acquired from the tail vein at 1-, 2-, or 4-week intervals for 70 weeks to estimate antizein IgG level.

This antigenicity study was further optimized with 600 µg of zein, and revealed that the zein-specific IgG started to increase significantly at week 4 and peaked at week 6 in serum for zein sizes of 250 nm, 450, 750, and 850 nm, as revealed by ELISA, where no distinct difference was found in the administered sizes, indicating no size-dependent effect following IM administration. Moreover, the administration of the 290-nm zein nanoparticles of different doses showed a peak IgG level at week 5 following the third inoculation, with a significantly higher titer with the 600 and 800 µg doses than the 200 µg, revealing the dose-dependent antigenic behavior of zein nanoparticles. The author also claimed a Th2-Type immune response caused by the different zein doses [139].

**Figure 11:**
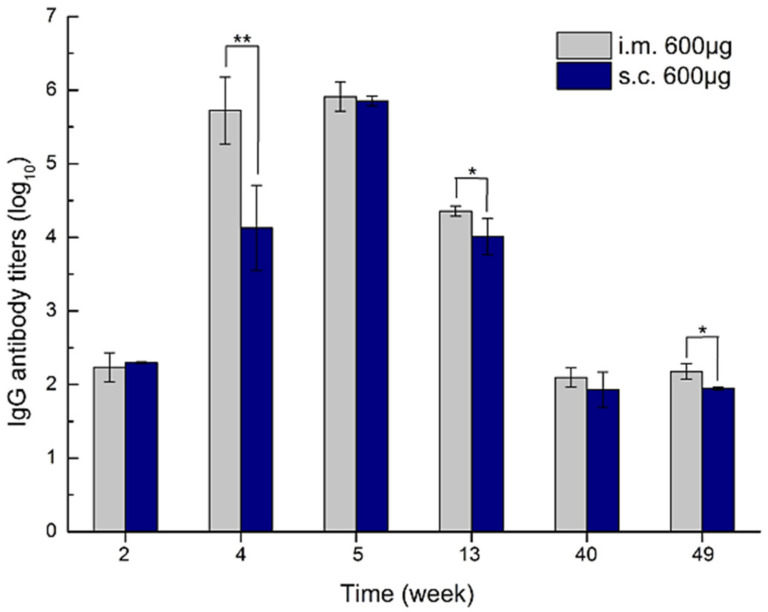
Zein-specific IgG titers in the sera of mice inoculated via intramuscular and subcutaneous routes with 600 µg of 290 nm zein particles at weeks 0, 1, and 3, respectively. The sera of all six mice in each group were separately evaluated three times by ELISA. Results are expressed as means of antibody titers calculated from six mice per group. All p-value calculations are based on the t-test of the two-sample equal variance hypothesis. * *p* < 0.05, and ** *p* < 0.01. Reproduced with permission from [139], International Journal of Nanomedicine, 2019.

The route of administration based on the 600 µg of the 290 nm zein nanoparticles demonstrated the same antigenic markers with both the IM and the SC routes. However, IM administration showed a more robust immune response than SC. Additionally, after day 7, more muscular inflammatory infiltration was observed in the injected muscles compared to the milder response obtained after SC injection (see Figure 11). Similar findings were observed by Hurtado-López [72] for IM injected zein microspheres.

**Figure 12:**
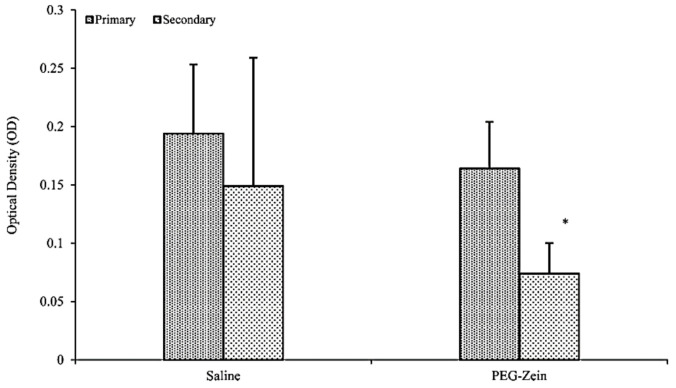
Antizein antibody levels after subcutaneous administration of mPEG−zein micelles in mice. The optical density of antizein antibodies in serum was measured after the third week of the first dose and the booster doses respectively. The results are represented as mean ± standard error of mean (*n* = 4). mPEG−zein micelles did not produce any antizein antibodies, and the values were similar to those of the saline control (* *p* > 0.05). Reproduced with permission from [13], American Chemical Society, 2012.

Protein PEGylation could be a unique method to reduce antigenicity. PEG coupling to several hydrophobic molecules tends to enhance the stealth properties in vivo and evade reticuloendothelial clearance [140]. Zein was, therefore, PEGylated and evaluated for in vivo antigenicity [13]. The results obtained from sandwich ELISA for the SC injected PEG-zein at a dose of 100 µg/50 mL saline revealed that the PEG-zein was nonimmunogenic and did not recall any antizein antibodies (see Figure 12). Comparatively, the oral administration of the PEGylated zein did not provoke any immune response. The author claimed that the PEGylation strategy and fabrication of the zein as a micellar system with smaller sizes could be helpful to avoid the uptake of zein by macrophages in vivo [13].

Lai and Guo [70] demonstrated the biodistribution profile of rhodamine B-loaded zein NPs after IV administration. The fluorescence signals of the administered nanoparticles in comparison to rhodamine solution were analyzed. Two hours postinjection, the results indicated a higher accumulation in the liver (56.7%), followed by the plasma (20.8%), spleen (7.3%), kidney (6.7%), lung (4.3%), and heart (4.1%). Compared to the free rhodamine solution, the maximum accumulation value was attained in the liver after 24 h for the zein nanoparticles where no fluorescence was detected for the rhodamine free solution at the same point. The diverse accumulation behavior of the nanoparticles was ascribed to the size of the designed nanoparticles, i.e., smaller than 200 nm, and according to the obtained results, the higher accumulation in the liver was attributed to the fenestrate associated cytoskeleton that controls the hepatic function of endothelial filtration and can be as large as 150 nm. Therefore, this could hinder the filtration of the particles and lead to accumulation in the liver. The author also suggested a size-dependent liver targeting of the designed nanoparticles.

In a recent study, Chen et al. [141] reported long-circulating zein-polysulfobetaine conjugate micelles for curcumin delivery. The study was based on the chemical conjugation of zein with a highly hydrophilic polymer, i.e., poly(sulfobetaine methacrylate), to form zein-based micelles. The micelles were investigated for their antigenicity and ability to alter macrophage shape, the primary defense guards for biological systems that show structural changes when influenced by an external antigenic stimulus. However, in comparison to the positive control effector and ordinary zein nanoparticles, the zein micelles showed no elongation of the macrophages. Instead, the spherical shape was conserved, like the shape of the negative control. In contrast, the ordinary zein and the positive control showed elongated shapes, indicating the activated macrophages. More interestingly, the in vivo circulation study of zein-based micelles loaded with Cy5.5 showed more intense fluorescence signals spreading over the whole body with a duration exceeding 72 h. Comparatively, the fluorescence signal in the free Cy5.5 group was reduced dramatically after 6 h and nearly vanished after 48 h, indicating a faster uptake of the noncoated zein nanoparticles by the reticuloendothelial system.

However, it is necessary to further explore the in vivo antigenicity of zein-based systems to further understand their in vivo behavior.

## 9. Concluding Remarks and Future Prospective

Based on the literature outlined in this review, it is evident that zein has been shown to be a biocompatible material in biomedical delivery applications. Despite its recognized drug-carrying capacity, most of the presented applications were restricted to oral delivery, with a few cases of topical application, such as wound healing scaffolds, in addition to a massive number of in vitro studies. However, a good understanding of its in vivo performance would open a new avenue of research in the biomedical field, including targeted drug delivery to treat a diverse range of pathological conditions. Hence, zein could be manipulated for the invasive in vivo administration of active moieties in cancer chemotherapy, or even for semi- or non- invasive routes such as photodynamic therapy in cancer or infectious conditions.

Moreover, tailoring the surface of the zein protein with various surface functionalities to specifically target cellular or subcellular localities is a unique strategy that could serve as a tool in the fight against gene-related disease conditions. Although folic acid and lactoferrin were employed as surface targeting probes in cancerous conditions, other probes like biotin and transferrin have not been fully explored to date. Additionally, the use of zein in inhalation therapeutics is worthy of study in cases that need localized administration and sustained release into the lung. Although there are limited reports on the application of zein-based systems to the lung, a good understanding of the compatibility of zein with the biological components of the lungs in distinct in vitro and in vivo models is worthy of consideration.

Compared to other nanoparticle platforms such as metallic and polymeric types, zein represents practicality in terms of its availability, low price, and ease of fabrication into a range of nanoparticulate designs. In addition, zein has been shown to be quickly engulfed by mammalian cells with negligible toxicity in several literature reports. Due to its amino acid composition, zein can undergo digestion in the liver following enteral or parenteral administration.

It may be concluded that optimal surface modification of the protein surface with a well-known biocompatible synthetic or biological polymer is a promising approach that should be investigated in order to extend the applicability of this cheap plant protein in the biomedical field.

## Figures and Tables

**Table 1:** The different zein fractions obtained from maize with their average molecular weights and percentages.

Protein Fraction	Average Molecular Weights (kDa)	Percentage	Reference
α-zein	19–22	up to 80%	[18,20,21,22]
β-zein	17–18	10–15%	[18,20,21]
γ-zein	27–28	5–10%	[18,21,22]
δ-zein	9–10	3%	[21]

**Table 2:** Different applications of zein-based systems presented in recent literature.

Zein Based Nanocomposite Platform	Application	Encapsulated Substance	Reference
zein/chitosan nano complex	oral drug and food delivery	curcumin and resveratrol	[97]
zein/carrageenan	oral drug delivery	curcumin and piperine coenzyme Q10 and piperine	[98,99]
hydrophilic whey isolate/zein	oral drug delivery	curcumin	[100]
zein/carrageenan/tween 80	drug delivery	curcumin	[101]
pectin from Akebia trifoliata var. Australis fruit peel and zein	drug and food delivery	curcumin	[102]
deamidated zein peptide	drug and food delivery	curcumin	[37]
zein/soybean polysaccharide	drug delivery	lutein	[103]
zein/caseinate	oral and parenteral drug delivery	simvastatin	[44]
zein/gold nanoparticles	antibacterial and larvicidal	the whole nanocomposite	[104]
zein/lactoferrin	drug delivery	7,8-dihydroxyflavone	[105]
zein/tea saponins	food and drug delivery	lutein	[106]
solid zein nanoparticles	drug delivery Gene delivery	maytansine PTEN and TRAIL genes	[107,108]
hollow zein nanoparticles	food and drug delivery	curcumin	[109]
zein/CMC	postharvest fruit preservation	natamycin	[56]
zein/bacterial cellulose	preservative and packaging	silymarin	[110]
zein/sodium deoxycholate	cytotoxic drug delivery	paclitaxel	[111]
lipid/zein core-shell nanocomposite	targeted pulmonary drug delivery	all-trans retinoic acid and genistein	[112]
mesoporous silica nanoparticles coated with zein polycarbolactone mixture	antimicrobial photodynamic therapy	methylene blue	[113]
zein/phosphatidylcholine hybrid nanoparticles	cancer photodynamic therapy	indocyanine green	[114]
zein/lactoferrin	functional food delivery	7,8-dihydroxyflavone	[105]
zein/hyaluronic acid	targeted drug delivery	curcumin honokiol	[115,116]
zein-cellulose nanocrystals core-shell nanoparticles	functional food delivery	curcumin	[117]
zein nanoparticles	oral drug delivery	resveratrol	[118]
polyamine modified zein	pest management	avermectin	[119]
zein/pluronic F68 nanoparticles	pest management	limonene and carvacrol	[120]
zein/fucoidan nanocomplex	oral drug delivery	curcumin	[121]
zein/sodium caseinate/fucoidan nanoparticles	functional food delivery	pterostilbene	[122]
zein/caseinate gelatin nanocomposite films	antifungal drug delivery	natamycin	[123]
lecithin/zein hybrid nanoparticles	oral drug delivery	panax notoginseng saponin	[124]
zein/PLGA nanoparticles	functional food delivery	docosahexaenoic acid	[125]
zein/carboxymethyl dextrin nanoparticles	oral drug delivery	curcumin	[126]
